# Comparison of Audiovisual and Paper-Based Materials for 1-Time Informed Consent for Research in Prison: A Randomized Clinical Trial

**DOI:** 10.1001/jamanetworkopen.2022.35888

**Published:** 2022-10-11

**Authors:** Stéphanie Baggio, Laurent Gétaz, Lauriane Giraudier, Lilian Tirode, Marta Urrutxi, Sonia Carboni, Aurore Britan, Robbie l’Anson Price, Hans Wolff, Patrick Heller

**Affiliations:** 1 Division of Prison Health, Geneva University Hospitals & University of Geneva, Geneva, Switzerland; 2 Institute of Primary Health Care (BIHAM), University of Bern, Bern, Switzerland; 3 Division of Tropical and Humanitarian Medicine, Geneva University Hospitals, Geneva, Switzerland; 4 Faculty of Medicine, University of Geneva, Geneva, Switzerland; 5 Clinical Research Center, Geneva University Hospitals, Geneva, Switzerland; 6 Geneva Finance Research Institute, University of Geneva, Geneva, Switzerland

## Abstract

**Importance:**

Few studies are available on informed consent (IC) among detained persons, even with ethics being a critical aspect of prison research. In IC research, audiovisual material seems to improve understanding and satisfaction compared with conventional paper-based material, but findings remain unclear.

**Objective:**

To compare audiovisual and paper-based materials for 1-time general IC for research in prisons.

**Design, Setting, and Participants:**

This cross-sectional randomized clinical trial was conducted in 2 corrections facilities in Switzerland (an adult prison and a juvenile detention center). The study was conducted from December 14, 2019, to December 2, 2020, in the adult prison and from January 15, 2020, to September 9, 2021, in the juvenile detention center. In the adult prison, study participation was offered to detained persons visiting the medical unit (response rate, 84.7%). In the juvenile detention center, all newly incarcerated adolescents were invited to participate (response rate, 98.0%).

**Interventions:**

Participants were randomized to receive paper-based conventional material or to watch a 4-minute video. Materials included the same legal information, as required by the Swiss Federal Act on Research Involving Human Beings.

**Main Outcomes and Measures:**

The main outcome was acceptance to sign the IC form. Secondary outcomes included understanding, evaluation, and time to read or watch the IC material.

**Results:**

The study included 190 adults (mean [SD] age, 35.0 [11.8] years; 190 [100%] male) and 100 adolescents (mean [SD] age, 16.0 [1.1] years; 83 [83.0%] male). In the adult prison, no significant differences were found between groups in acceptance to sign the IC form (77 [81.1%] for paper-based material and 81 [85.3%] for audiovisual material; *P* = .39) and to evaluate it (mean [SD] correct responses, 5.09 [1.13] for paper-based material and 5.01 [1.07] for audiovisual material; *P* = .81). Understanding was significantly higher in the audiovisual material group (mean [SD] correct responses, 5.09 [1.84]) compared with the paper-based material group (mean [SD] correct responses, 4.61 [1.70]; *P* = .04). In the juvenile detention center, individuals in the audiovisual material group were more likely to sign the IC form (44 [89.8%]) than the paper-based material group (35 [68.6%], *P* = .006). No significant difference was found between groups for understanding and evaluation. Adults took a mean (SD) of 5 (2) minutes to read the paper material, and adolescents took 7 (3) minutes.

**Conclusions and Relevance:**

Given the small benefit of audiovisual material, these findings suggest that giving detained adults and prison health care staff a choice regarding IC material is best. For adolescents, audiovisual material should be provided. Future studies should focus on increasing understanding of the IC process.

**Trial Registration:**

ClinicalTrials.gov Identifier: NCT05505058

## Key Points

**Question:**

Is audiovisual material better than paper-based material for 1-time general informed consent in prison research?

**Findings:**

In this randomized clinical trial of 190 adults in prison and 100 adolescents in juvenile detention, 85.3% of adults and 89.8% of adolescents signed the informed consent form after seeing a video on informed consent, whereas 81.1% of adults and 68.6% of adolescents signed informed consent forms after reading the paper-based material.

**Meaning:**

Given the small benefit of audiovisual material in this study, detained adults and prison health care staff should have a choice regarding informed consent material and detained adolescents should be provided with audiovisual material.

## Teaser

This randomized clinical trial assesses whether audiovisual or paper-based informed consent material was more likely to result in research acceptance by individuals in prison.

## Introduction

People living in detention (PLD^1^) constitute a vulnerable population with a heavy burden of diseases.^2,3^ They also often lack access to adequate health care, resulting in important health disparities compared with the general population.^4,5^ Because of past abusive research practices, PLD now benefit from ethical protective measures.^6^ However, these measures reduced the amount of research conducted among PLD, thus violating the principle of equivalence of care.^7,8^ This principle means that prison health services must reach a standard equivalent of care as those provided to the wider community. If PLD are excluded from research, they may also be excluded from research benefits. Research to understand and improve the health of PLD is needed and should be considered as an ethical principle.^4,5,8^

People living in detention often have a low educational level and health literacy, language barriers, and health-related problems that may affect cognition.^4^ Therefore, informed consent (IC) is a challenge, and research is needed to ensure that research participation is ethically conducted.^9^ However, few studies are available on understanding of IC among PLD.^4^ Previous studies reported that less than half of PLD correctly answered questions about IC^4^ and that even if almost all PLD had an adequate capacity to consent, they had a significantly lower ability to understand consent material compared with control individuals from the general population.^10^ Even in the general population, IC is a challenging issue.^11,12^

To deal with these critical issues, different supports for IC have been tested. In clinical practice, audiovisual material enhanced immediate information recall, without sufficient evidence of late recall, anxiety, and satisfaction.^13^ In research, it seems that audiovisual material improved understanding and satisfaction compared with conventional paper-based IC, but findings remain unclear.^14,15^ Studies with robust methods are still needed to achieve a better understanding of the benefits of audiovisual material.^14^ Because PLD are likely to be illiterate,^4^ audiovisual material could represent a valid alternative to conventional paper-based IC. This randomized clinical trial conducted in PLD aimed to fill in this gap and compared audiovisual and paper-based materials for 1-time general IC for research.

## Methods

### Design and Setting

This cross-sectional randomized clinical trial had a parallel randomized design (allocation 1:1) and took place in 2 prisons in Geneva, Switzerland. Champ-Dollon (adult prison) is the largest pretrial prison in Switzerland, with a capacity of 398 places for adult PLD. La Clairière (juvenile detention center) has a capacity of 30 places for youths aged 10 to 18 years. Both prisons have a medical unit. The study was conducted between December 14, 2019, and December 22, 2020, in the adult prison and between January 15, 2020, and September 9, 2021, in the juvenile detention center. The study was performed in accordance with the Declaration of Helsinki.^16^ Geneva’s Cantonal Ethics Committee approved the study protocol. Because the outcome of the study was to provide IC, a written IC was not used before study participation in order to not create bias. Participants provided oral consent to participate in the study. The study followed the Consolidated Standards of Reporting Trials (CONSORT) reporting guideline for randomized clinical trials. The trial was retrospectively registered on ClinicalTrials.gov. It was first intended as an exploratory study and, accordingly, was not registered. The trial protocol can be found in Supplement 1.

### Participants

In the adult prison, 228 men were invited to participate, with 193 accepting (response rate, 84.7%). Three men dropped out after randomization because they were not interested anymore, leaving a final sample of 190 (95 in each group). In the juvenile detention center, 102 adolescents were invited to participate and 2 declined participation (response rate, 98.0%), leaving a final sample of 100 (51 in the paper-based material group and 49 in the audiovisual material group). The study flow chart is presented in the eFigure in Supplement 2. Exclusion criteria included severe acute psychiatric issues that did not allow for IC, being younger than 18 years in the adult prison, and being younger than 14 years in the juvenile detention center.

### Procedures

In the adult prison, study participation was offered to PLD visiting the medical unit (approximately 75% of all PLD). They were invited while they were in the waiting room after a medical visit. In the juvenile detention center, all newly incarcerated adolescents were invited to participate until the desired sample size was reached. Participants were interviewed in a room of the medical unit. Participants watched or read the information for IC and could ask any question about it. They were then invited to sign the IC form and complete a 15-minute face-to-face questionnaire.

The study description and all study documents (written IC form, video, and questionnaire) were provided in 11 languages: Albanian, Arabic, English, French, Georgian, German, Italian, Portuguese, Romanian, Russian, and Spanish. Questionnaires were translated from English and then back-translated, with resolution of discrepancies. No PLD declined participation because of language barriers.

### Materials for Informed Consent

Participants were randomized to 2 groups: paper-based conventional material or a 4-minute video. Materials included the same legal information, as required by the Swiss Federal Act on Research Involving Human Beings. The IC did not deal with a specific study but was a 1-time general IC for research.^17^ This general IC allows using routinely collected clinical and biological data from the hospital’s medical files for past, present, and future research. Research projects using these data must nonetheless be submitted to the Cantonal Ethics Committee. The English versions of the video and written material are available in the eAppendix in Supplement 2. The written material was developed by the Swiss Association of Research Ethic Committees, and the booklet was designed by the Clinical Research Center of the Geneva University Hospitals to be used in the whole hospital. The video was developed by a science filmmaker for the project’s purposes.^18^ After reading or seeing the material for IC, participants could ask questions without a time limit. The interviewers were trained to provide relevant information and to answer questions about the general IC.

### Measures

The primary outcome was acceptance to sign the IC form. Secondary outcomes included understanding of the IC, evaluation of the IC, and time needed to read or watch materials. We also explored which sociodemographic and clinical factors were associated with understanding of the IC.

To assess understanding of the IC, we self-developed 8 questions related to the understanding of the IC (true or false questions). The questionnaire is available in the eAppendix in Supplement 2. Questions were developed in collaboration with a team involved in IC research at the University of Geneva and pretested in the study population. We computed a sum score of correct answers, ranging from 0 to 8. To assess evaluation of the IC, we self-developed 9 questions related to the evaluation of the IC, assessed on a 6-point scale (eAppendix in Supplement 2). Questions were developed with the same team as described above and pretested. We computed a mean score (Cronbach α = 0.83). For time spent reading or watching the IC material, we assessed how long participants took to read the paper-based material. The video had a unique length of 4 minutes.

Participants were offered study material in Albanian, Arabic, English, French, Georgian, German, Italian, Portuguese, Romanian, Russian, and Spanish. We used a binary variable of French (the language spoken in this French-speaking part of Switzerland) vs other languages. We used the Short Test of Functional Health Literacy to assess health literacy.^19^ This test is composed of 3 questions assessed on a 6-point scale. Because there was a cell effect, we used 2 categories: high health literacy (mean score ≥5) vs low or moderate health literacy (mean score <5).

Other variables of interest included clinical information and sociodemographic variables. Participants provided information on psychiatric diagnoses, somatic illnesses, and use of any medications (coded as presence or absence). We also assessed age, level of education (primary vs secondary or tertiary), legal residence in Switzerland (yes vs no), health insurance (yes vs no), and duration of incarceration (<6 months vs ≥6 months for adults and <2 weeks vs ≥2 weeks for adolescents). Sex was assessed in the juvenile detention center (the adult prison only held male PLD).

### Statistical Analysis

A simple random allocation was used in each center because the risk of significant imbalance was negligible with the expected sample size.^20^ A random allocation sequence was generated with the software R, version 3.6.1 (R Foundation for Statistical Computing). Three trained interviewers (2 for the adult prison [L.G. and M.U.] and 1 for the juvenile detention center [L.T.]) enrolled participants and assigned them to 1 group without blinding. We computed a sample size of 190 (95 in each group), with α = .05, power = 0.80, and estimated percentages of signed IC of 50% for paper and 70% for video.^21^ The sample size was computed for the adult prison. The juvenile detention center was included for exploratory purposes. We computed a sensitivity power analysis to assess the minimum effect size we could detect in the juvenile detention center.

We computed descriptive statistics for all variables. We controlled whether the randomization worked, comparing groups (paper or audiovisual material) for all variables using logistic regressions (except for age [linear regression]). We then tested the relationships between groups and (1) signing, (2) understanding, and (3) evaluating the IC. We then presented unadjusted and adjusted logistic (signing) and linear (understanding and evaluation) regression models. The adjusted analyses controlled for all covariates to improve precision and power and were considered as primary analyses.^22^ Finally, we performed a multiple linear regression to identify factors associated with understanding the IC.

In the sensitivity power analysis, with sample sizes of 49 in the audiovisual material group and 51 in the paper-based material group, α = .05, power = 0.80, and a 1-tailed independent *t* test, the effect size was *d* = 0.50. Therefore, the study was powered to detect medium effect size in the juvenile detention center.

Analyses were performed as intention-to-treat using Stata software, version 17 (StataCorp LLC) separately for each prison and with GPower, version 3.1.9.4 (Foshan G-power Technology Co Ltd) for the sensitivity power analysis.

## Results

The study included 190 adults (mean [SD] age, 35.0 [11.8] years; 190 [100%] male) and 100 adolescents (mean [SD] age, 16.0 [1.1] years; 83 [83.0%] male and 17 [17.0%] female) participants. Descriptive statistics and comparisons between groups are reported in Table 1 for the adult prison and in Table 2 for the juvenile detention center. Main results are reported in Table 3. Overall, 158 adults (83.2%) and 79 adolescents (79.0%) signed the IC form. In the adult prison, no significant differences were found between groups in acceptance to sign the IC form (77 [81.1%] for paper-based material and 81 [85.3%] for audiovisual material; *P* = .39) and to evaluate it (mean [SD] correct responses, 5.09 [1.13] for paper-based material and 5.01 [1.07] for audiovisual material; *P* = .81).

**Table 1.** Descriptive Statistics for the Incarcerated Individuals in the Adult Prison and Comparisons Between Types of Informed Consent^a^

Variable	Overall (N = 190)	Type of informed consent		*P* value
		Paper (n = 95)	Audiovisual (n = 95)	
Age, mean (SD), y	35.0 (11.8)	35.3 (12.5)	34.8 (11.1)	.75
Sex				
Male	190 (100)	95 (100)	95 (100)	NA
Female	0	0	0	
Secondary or tertiary level of education^b^	164 (87.2)	83 (89.3)	81 (85.3)	.42
French language	110 (57.9)	56 (59.0)	54 (43.2)	.77
Legal residence in Switzerland^c^	81 (43.8)	48 (51.6)	33 (35.9)	.03
Have a health insurance^b^	94 (50.0)	54 (58.1)	40 (42.1)	.03
Duration of incarceration ≥6 mo^c^	68 (63.2)	37 (40.2)	31 (33.3)	.33
High health literacy^b^	110 (58.5)	58 (62.4)	52 (54.7)	.29
Presence of a psychiatric diagnosis^d^	38 (20.1)	19 (20.2)	19 (20.0)	.97
Presence of a somatic illness^d^	75 (39.7)	38 (40.4)	37 (38.0)	.84
Use of any medication^d^	126 (66.7)	64 (68.1)	62 (65.3)	.68

Abbreviation: NA, not applicable.

^a^ Data are presented as number (percentage) of study participants unless otherwise indicated. All data are from logistic regressions except for age, which is from linear regression.

^b^ Data missing for 2 individuals.

^c^ Data missing for 5 individuals.

^d^ Data missing for 1 individual.

**Table 2.** Descriptive Statistics for Incarcerated Individuals in the Juvenile Detention Center and Comparisons Between Types of Informed Consent^a^

Variable	Overall (N = 100)	Type of informed consent		*P* value
		Paper (n = 51)	Audiovisual (n = 49)	
Age, mean (SD), y	16.0 (1.1)	16.1 (1.1)	15.9 (1.0)	.25
Sex				
Male	83 (83.0)	42 (82.4)	41 (83.7)	.86
Female	17 (17.0)	9 (17.7)	8 (16.3)	
Secondary or tertiary level of education	90 (90.0)	47 (92.2)	43 (87.8)	.47
French language	86 (86.0)	44 (86.3)	42 (85.7)	.94
Legal residence in Switzerland	80 (80.0)	43 (84.3)	37 (75.5)	.27
Have health insurance	84 (84.0)	43 (84.3)	41 (83.7)	.93
Duration of incarceration ≥2 wk	37 (37.0)	18 (35.3)	19 (38.8)	.72
High health literacy^b^	63 (63.6)	30 (58.8)	33 (68.8)	.31
Presence of a psychiatric diagnosis^c^	16 (16.7)	10 (20.4)	6 (12.8)	.32
Presence of a somatic illness	31 (31.0)	14 (27.5)	17 (34.7)	.43
Use of any medication	40 (40.0)	19 (37.3)	21 (42.9)	.57

^a^ Data are presented as number (percentage) of study participants unless otherwise indicated. All data are from logistic regressions except for age, which is from linear regression.

^b^ Data are missing for 1 individual.

^c^ Data are missing for 4 individuals.

**Table 3.** Associations Between Type of Informed Consent (Paper or Audiovisual) and Outcomes

Variable	Paper	Audiovisual	Unadjusted		Adjusted^a^	
			Estimate (95% CI)	*P* value	Estimate (95% CI)	*P* value
**Adult prison**						
Consent^b^	77 (81.1)	81 (85.3)	1.35 (0.63 to 2.91)	.44	1.46 (0.61 to 3.50)	.39
Understanding^c^	4.61 (1.70)	5.09 (1.84)	0.48 (−0.02 to 0.99)	.06	0.60 (0.11 to 1.09)	.04
Evaluation^c^	5.09 (1.13)	5.01 (1.07)	−0.08 (−0.40 to 0.23)	.61	−0.03 (−0.32 to 0.25)	.81
**Juvenile detention center **						
Consent^b^	35 (68.6)	44 (89.8)	4.02 (1.34 to 12.06)	.01	6.06 (1.68 to 21.91)	.006
Understanding^c^	6.02 (1.82)	6.31 (1.37)	0.29 (−0.35 to 0.93)	.38	0.45 (−0.11 to 1.01)	.11
Evaluation^c^	5.19 (0.76)	5.39 (0.73)	0.20 (−0.10 to 0.50)	.18	0.19 (−0.09 to 0.47)	.18

^a^ Analyses for covariates (age, sex [in the juvenile detention center], level of education, language, legal residence in Switzerland, health insurance, health literacy, psychiatric diagnosis, somatic illness, and medication).

^b^ Number (percentage) of study participants with odds ratios.

^c^ Means (SDs) with b estimates.

Understanding was significantly higher in the audiovisual material group compared with the paper-based material group. In the juvenile detention center, the audiovisual material group was more likely to sign the IC form than the paper-based material group. No difference was found between groups for understanding and evaluation. Adults took a mean (SD) of 5 (2) minutes to read the paper-based material, and adolescents took 7 (3) minutes.

Regardless of the type of material, participants had a mean (SD) of 4.8 (1.8) correct answers in the adult prison (9 [4.7%] answered the 8 questions correctly) and 6.2 (1.6) in the juvenile detention center (22 [22.0%] answered the 8 questions correctly). Table 4 reports descriptive results for understanding of the IC.

**Table 4.** Descriptive Statistics for Understanding and Evaluation of the Informed Consent

Variable	Adult prison (n = 190)	Juvenile detention center (n = 100)
**Understanding, No. (%)**		
My name is kept with my data (F)	125 (65.0)	54 (54.0)
My decision is valid indefinitely (T)	118 (62.1)	75 (75.0)
I must justify my decision if I refuse to participate (F)	88 (46.3)	84 (84.0)
My data can be sold (F)	148 (77.9)	92 (92.0)
My data may be used elsewhere than in Geneva (T)	124 (65.3)	72 (72.0)
I can withdraw my consent at any time (T)	155 (81.6)	82 (82.0)
If I accept, the doctors will do unnecessary additional tests for my medical follow-up (F)	69 (36.3)	74 (74.0)
Only authorized scientists will have access to my data (T)	155 (81.6)	83 (83.0)
**Evaluation, mean (SD)**		
The consent is easy to understand	4.7 (1.44)	4.8 (1.28)
The information provided is sufficient	4.6 (1.51)	4.9 (1.78)
You felt completely free to sign the consent	5.6 (1.11)	5.5 (1.03)
You did not fear at all that your decision would affect your access to care	5.2 (1.49)	5.6 (1.12)
You did not fear at all that your decision would influence your legal proceedings	5.3 (1.47)	5.7 (0.98)

Abbreviations: F, false; T, true.

Table 5 reports the factors associated with the understanding of the IC (number of correct answers), without considering the type of material. In the adult prison, being younger, speaking French, legally residing in Switzerland, and having high health literacy were associated with a significantly higher number of correct answers compared with being older, not speaking French, not legally residing in Switzerland, and having low to moderate health literacy. In the juvenile detention center, speaking French, being female, legally residing in Switzerland, having high health literacy, and taking no medication were associated with a higher number of correct answers compared with not speaking French, being male, not legally residing in Switzerland, having low to moderate health literacy, and taking medication.

**Table 5.** Multiple Linear Regressions of Factors Associated With Understanding of the Informed Consent (Number of Correct Answers)^a^

Variable	Adult prison		Juvenile detention center	
	Estimate (95% CI)	*P* value	Estimate (95% CI)	*P* value
Age	−0.02 (−0.05 to −0.01)	.05	−0.12 (−0.42 to 0.17)	.40
Male sex	NA	NA	−1.03 (−1.81 to −0.24)	.01
Secondary or tertiary level of education	0.20 (−0.58 to 0.98)	.61	0.03 (−1.08 to 1.14)	.96
French language	0.58 (0.01 to 1.16)	.05	1.07 (0.09 to 2.04)	.03
Legal residence in Switzerland	0.72 (0.12 to 1.32)	.02	1.47 (0.43 to 2.51)	.006
Have health insurance	−0.06 (−0.68 to 0.54)	.84	−0.04 (−1.22 to 1.15)	.95
Duration of incarceration ≥6 mo for adults and ≥2 wk for juveniles	0.23 (−0.29 to 0.76)	.38	0.69 (−0.03 to 1.40)	.06
High health literacy	0.72 (0.17 to 1.28)	.01	0.69 (0.07 to 1.32)	.03
Presence of a psychiatric diagnosis	0.32 (−0.33 to 0.96)	.34	0.47 (−0.32 to 1.27)	.24
Presence of a somatic illness	0.24 (−0.29 to 0.76)	.38	0.42 (−0.24 to 1.09)	.21
Use of any medication	−0.02 (−0.58 to 0.55)	.96	−0.75 (−1.44 to −0.07)	.03

Abbreviation: NA, not applicable.

^a^ Example of interpretation: When the age increased by 1 year, there was a 0.02-point decrease on the number of correct answers.

## Discussion

In this randomized clinical trial, findings for the primary outcome, signing the IC form, were inconsistent across prisons. In the adult prison, PLD were similarly likely to sign the IC, whatever material was used. In the juvenile detention center, PLD were more likely to sign the IC after watching the video than after reading the paper-based material. Adult results were in line with previous findings reporting no effect of video vs paper-based material on acceptance to sign IC forms and enroll in studies in the general population.^14,15^ Adolescents spend more time on digital media than reading,^23^ which might explain why there was a preference for the audiovisual material in adolescents compared with adults. This outcome confirmed the findings of a previous study^24^ in which audiovisual interventions have been demonstrated to be superior to other interventions among adolescents (eg, on knowledge of HIV and consent to HIV testing).

Findings for the first secondary outcome, understanding the IC, were also inconsistent across prisons. Although understanding was slightly better with the audiovisual material in the adult prison, no statistically significant difference was found between audiovisual and paper-based material in the juvenile detention center. These results are in line with previous studies^14,15^ that reported a small positive effect of video on understanding the IC. Of note, the study was not powered enough to detect small effect sizes in the juvenile detention center.

For the secondary outcome of evaluation of the IC, no effect was found of the type of material on IC evaluation. The overall rating was high, meaning that the evaluation was good for both materials in both prisons. This finding suggests good acceptability of the IC in PLD.

Finally, no notable difference was found in time spent reading or watching the IC material. Compared with the 4-minute video, adults spent a mean (SD) of 5 (2) minutes reading, and adolescents spent 7 (3) minutes.

Overall, our study highlights a small influence of audiovisual material to improve IC compared with paper-based material in PLD, a vulnerable population with low levels of education, limited health literacy, language barriers, and potential cognitive problems.^4^ Evaluations were high for both types of materials, suggesting good acceptability, and no notable gain in time was seen with the audiovisual material. We therefore suggest giving adult PLD and prison health care staff the choice between audiovisual and paper-based material. For adolescents, we recommended the audiovisual material.

Of note, the overall acceptance rates were high in both prisons: 82.3% in the adult prison and 79.0% in the juvenile detention center. These acceptance rates suggest that PLD were not reluctant to contribute to research. However, understanding could be improved. Indeed, adults had a mean of only 4.8 correct answers, and 4.7% answered all questions correctly. Results were better for adolescents (6.2 correct answers and 22.0% answering all questions correctly) but could also be improved. In both prisons, characteristics associated with lower understanding were language barriers, having an illegal residence in Switzerland, and low to moderate health literacy. Older age in the adult prison and being male in the juvenile detention center were also associated with lower levels of understanding. Similar subgroups have already been identified in previous research^4,10^ concluding that IC is not well understood by PLD. Special attention is needed for these subgroups to protect them from unethical research and to make them aware of their rights. Some guidelines have already been proposed to improve IC. For example, the teach-to-goal consent method has been tested in PLD.^4^ This process includes a comprehension test and retest after feedback if needed to achieve a voluntary and truly informed IC process.

The 1-time general IC for research allows easier access to large sample sizes, thus enabling a better understanding of the characteristics and health needs of PLD. It may thus help tackle health (documentation) inequalities.^5^ Finally, it allows a better control and respect of legal ethics norms about data reuse, a critical issue in prison populations, in which ethical principles must be scrupulously respected. Therefore, the 1-time general IC constitutes a fair balance between neglect of a vulnerable population and risk of exploitation.^9^

### Limitations

This study nonetheless has some limitations. A first limitation was that the study took place at the medical prison unit in the adult prison, meaning that PLD who did not seek health care were not included. These PLD could include those without health problems or vulnerable PLD not willing to seek health care (eg, because of language barriers or specific psychiatric disorders). A second limitation was that some PLD refused to participate in the adult prison, which might have inflated the acceptance rate to sign IC forms. However, because the study had a randomized design, this refusal of participation probably affected both groups (audiovisual and paper-based materials) similarly. A third shortcoming was that we relied on self-reports, which limited the reliability of clinical information, especially for psychiatric disorders, which might have been underreported. In addition, we used self-developed questionnaires to assess understanding and evaluation of the IC, meaning that these questionnaires were not validated. Fourth, we did not ask about the native language of participants and how fluent they were in the language they chose for the study material, which limited our findings’ understanding. A fifth shortcoming was that we could not assess inter-interviewer reliability because interviewers did not get responses from the same participants. A sixth limitation was that the study was powered to detect medium effect sizes in the juvenile detention center. Nonsignificant results should therefore be interpreted cautiously. Seventh, some limitations are related to the IC itself. The material used for the paper-based vs audiovisual IC was not exactly the same. Although the paper material described in a dry language how physicians, scientists, and patients could contribute to research, the video material used a very sick patient as an example. These differences could partially explain the different rates of consent in the juvenile sample. In addition, our study compared paper-based and audiovisual formats for a general 1-time IC; therefore, results may not be applicable to IC designed for participation in a specific study. Comparison of different formats and evaluation of understanding of the IC may be a relevant prestudy process, for example, before starting a large randomized clinical trial (eg, using the teach-to-goal process described above).

## Conclusions

In this randomized clinical trial, audiovisual material to obtain IC appeared to be slightly more effective than paper-based material. Given the small benefit of this material for PLD, the choice of the material should be left to adult PLD and prison health care staff, and audiovisual material should be offered to adolescents. One-time IC may improve equivalence of health care in prison and reduce health disparities, but future studies should focus on increasing understanding of the IC process.

## Notes

Supplement 1.
Trial Protocol and Statistical Analysis Plan

Supplement 2. **eFigure.** Flowchart **eAppendix.** Information on Informed Consent

Supplement 3.
Data Sharing Statement
