# Supplementary material for: Comparison of Audiovisual and Paper-Based Materials for 1-Time Informed Consent for Research in Prison: A Randomized Clinical Trial
Source: JAMA Netw Open. 2022 Oct 11;5(10):e2235888. doi: 10.1001/jamanetworkopen.2022.35888 (PMC9554696; doi:10.1001/jamanetworkopen.2022.35888)
Supplement: Supplement 1. — Trial Protocol and Statistical Analysis Plan [file jamanetwopen-e2235888-s001.pdf]

One-time informed consent for research in prison: A randomized comparison between audio-visual and written materials

Research legislation:	Ordinance on human research with the exception of Clinical trials (HRO) (1).
Type of Research Project:	Research project involving human subjects
Risk Categorisation:	Risk category A
Project Leader:	Dr. Stéphanie Baggio,<br>Division of Prison Health, Geneva University Hospitals, chemin du Petit Bel Air 2, 1226 Thônex, Suisse<br>stephanie.baggio@hcuge.ch<br>022 305 52 18

PROTOCOL SIGNATURE FORM

Study Title	One-time informed consent for research in prison: A randomized comparison between audio-visual and written materials

The project leader has approved the protocol version ***2 (dated 17.10.2019)**,* and confirms hereby to conduct the project according to the protocol, the Swiss legal requirements (1)], current version of the World Medical Association Declaration of Helsinki (2) and the principles of Good Clinical Practice.

Project leader:

Site : *Geneva University Hospitals*

Name: Stéphanie Baggio, Laurent Gétaz (Champ-Dollon), Patrick Heller (La Clairière)

Date: 17.10.2019 _________ Signature:

TABLE OF CONTENTS

TABLE OF CONTENTS 3
GLOSSARY OF ABBREVATIONS 4
1 BACKGROUND AND PROJECT RATIONALE 5
2 PROJECT OBJECTIVES AND DESIGN 6
2.1 Hypothesis and primary objective 6
2.2 Primary and secondary endpoints 6
2.3 Project design 6
3 PROJECT POPULATION AND STUDY PROCEDURES 6
3.1 Project population, inclusion and exclusion criteria 6
3.2 Recruitment, screening and informed consent procedure 7
3.3 Study procedures 7
3.4 Withdrawal and discontinuation 7
4 STATISTICS AND METHODOLOGY 8
4.1. Statistical analysis plan 8
4.2. Handling of missing data 8
5 REGULATORY ASPECTS AND SAFETY 8
5.1 Local regulations / Declaration of Helsinki 8
5.5 Amendments 8
5.6 End of project 8
6 FURTHER ASPECTS 9
6.1 Overall ethical considerations 9
6.2 Risk-Benefit Assessment 9
6.3 Rationale for the inclusion of vulnerable participants 9
7 QUALITY CONTROL AND DATA PROTECTION 10
7.1 Quality measures 10
7.2 Data recording and source data 10
7.3 Confidentiality and coding 10
7.4 Retention and destruction of study data and biological material 10
8 FUNDING / PUBLICATION / DECLARATION OF INTEREST 10
9 REFERENCES 10
10 ANNEXE : LANGUES PARLÉES À CHAMP-DOLLON**ERROR! BOOKMARK NOT DEFINED.**

GLOSSARY OF ABBREVATIONS

BASEC	*Business Administration System for Ethical Committees*
CRF	*Case report form*
FOPH	*Federal Office of Public Health*
HRA	*Human Research Act*
HRO	*Ordinance on Human*
HUG	*Geneva University Hospitals*
S-TOFHLA	*Short Test of Functional Health Literacy*

1 BACKGROUND AND PROJECT RATIONALE

1.1 General consent for research at HUG

The HUG proposes a general consent for research which allows the re-use of medical data and residual biological material of patients collected during their (past, present, and future) care. The introduction of this general consent for research is part of the strategic project vision 20/20, which supports and promotes research activities at the HUG. It has been implemented in several departments and 32,000 patients have already given their consent, with an acceptance rate of 92%.

There is an explanatory brochure presenting the reasons and usefulness of consent and addressing questions related to data protection and use, accompanied by the consent form. Acceptance and signature of the consent form allows the re-use of clinical and biological data in a coded form for research purposes.

1.2 Use of the general consent for research in the Division of prison health

The team of the HUG prison health service wishes to implement general consent for research in its service, which provides medical follow-up for incarcerated persons. Persons detained in Geneva are considered as patients of the HUG, in the context of professional independence (the nursing staff is attached to the general health system and therefore to the HUG, not to the prison administration) benefiting from the equivalence of care (same care as that available to the general population). The legal service of the HUG has given an agreement in principle for the deployment of this project within the service (request made by the Clinical Research Centre). Nevertheless, detained and under-aged patients constitute a particular population, very vulnerable and perhaps reluctant to share their medical data. In particular, it is crucial to ensure that these persons, deprived of their liberty, understand that their consent is voluntary and that a refusal will not have any consequence on their treatment or care (3). In other words, it is necessary to ensure that consent is informed. In a historical context of non-ethical research using detained persons (see for example 4), this is a crucial issue. However, while detained persons are now protected from various forms of abuse, this may have had the consequence of reducing research involving them (5), to the detriment of understanding their characteristics and vulnerabilities.

A general consent for research will encourage research on prison populations by facilitating access to their medical data in order to study and reduce health disparities (6), for this population with multiple somatic and psychiatric comorbidities (7-9).

1.3 Material for informed consent

Moreover, the question of a paper-based material is also questionable. Detained persons may have reading and low literacy (3), which may reduce the likelihood of consent and especially informed consent. About 20% of detained persons are illiterate, making it difficult to use paper-based materials. This is a potential weakness of the general consent for research as currently proposed by the HUG: It may exclude vulnerable populations, who are less likely to accept. This procedure could therefore, in the long run, reinforce inequalities in the documentation of health status, while the most vulnerable also have poorer health. Video consent would be a more appropriate method for vulnerable populations. While it appears to be effective in obtaining informed consent in the general population (10, to our knowledge it has not been tested in prisons.

Thus, despite these major issues, ethical research on detained persons remains limited (3, 5) and this study aims to fill in this research gap. To the best of our knowledge, no study on the informed consent of detained persons has been published in Switzerland.

2 PROJECT OBJECTIVES AND DESIGN

2.1 Hypothesis and primary objective

The objective of this project is to test whether the use of general consent for research at the HUG is feasible in the Division of prison health and to obtain an estimate of the acceptance rate as well as the characteristics of those who refuse. This is an exploratory study which aims to answer the following questions:
Q1. What is the acceptability rate of general consent for research in detained persons?
Q2. What are the characteristics (socio-demographic and medical) of detained person who refuse to give their consent?
Q3. Which material (paper-based or video) is more effective?

2.2 Primary and secondary endpoints

The primary outcome is whether participants sign the inform consent.

In addition, participants will complete a questionnaire including the following scales:
1. Literacy: French version of the Short Test of Functional Health Literacy (S-TOFHLA) (10).
2. Questions evaluating the consent, measured on 6-point Likert scales. These questions were developed in collaboration with the project team "Patients' views and institutional requirements for information and consent documents in medical research" of the University of Geneva.
3. Questions assessing the understanding of the consent, assessed with true/false answers.

Socio-demographic and medical information will also be collected:
- Socio-demographic characteristics: Age, region of origin, arrival in Switzerland and permit in Switzerland, education, health insurance, date of entry in prison.
- Health: Medical consultations before and during detention (nurses, doctors and psychologists/psychiatrists), diagnosed somatic diseases and psychiatric disorders, past and current treatments.
For participants who consent to the use of their medical data, medical information will also be collected directly by the interviewer from the participant's medical file (somatic diseases, psychiatric disorders, and current treatments).

2.3 Project design

This is an exploratory randomized cross-sectional trial. This project will be conducted at the Geneva pre-trial prison (Champ-Dollon) and at the juvenile detention center (La Clairière).
We will attribute participants randomly in one of the two groups, stratified on the prison:
1) The first group will read the paper-based material;
2) The second group will watch the video.

3 PROJECT POPULATION AND STUDY PROCEDURES

3.1 Project population, inclusion and exclusion criteria

Two samples will be used for this study.
The first is composed of adult males detained in the adult prison (n=1766 entries in 2017) (n=190).
The second is composed of adolescents incarcerated in the juvenile detention center (n=100).
The inclusion criteria are: 1) to be ≥18 years old for the adult prison and ≥14 years old for the juvenile detention center, 2) to be able to communicate in one of the languages of the study material (see list of the most spoken languages at the adult prison in the appendix), 3) to agree to participate in this study.

The only exclusion criterion is the presence of an acute psychiatric problem preventing the person from participating in the study.

3.2 Recruitment, screening and informed consent procedure

In both prisons, the study will take place in the prison medical unit. In the adult prison, approximately 75% of detained persons visit the medical unit (100% in the juvenile detention center).
As the study is about consent, no specific consent is requested for this study (see section 6.1).
Detained persons will be informed that their participation in the study is voluntary, independent of their medical care and legal proceedings, and that they can withdraw at any time.

3.3 Study procedures

Participants will be included for three months (November 2019 to January 2020) in the adult prison and for one year (November 2019 to October 2020) in the juvenile detention center[1].
In the adult prison, detained person going to the medical unit will be proposed to participate in the study after their consultation. The interviewer (doctor, medical student or psychologist) will briefly explain the project. The participants will be informed that they will be asked questions related to their health and medical follow-up.
In the juvenile detention center, all minors will be invited to participate in the study by a psychologist.
Those who agree to participate will read the paper-based material or watch the video. The doctor will allow sufficient time to read the booklet/watch the video and will be available to answer questions and ensure that the participant has understood the principle of consent and the information given. Each participant will then be invited to sign the general informed consent.
All participants will answer questions about their socio-demographic data, somatic and psychiatric illnesses and medical consultations, health literacy, and about the consent itself (evaluation and understanding).
Questions will be asked by the interviewer who will enter the answers directly into a computer.

The estimated time per participant is 15 minutes to propose/explain the project, read the paper-based material/watch the video, and obtain consent or not and 15 minutes to complete the questionnaire.

The main risk of the study is that a high number of people refuse to participate in the study. To avoid this risk of a poorly representative sample, participation in the study will be remunerated with a gift voucher for the Payot bookshop (juvenile detention center) or a grocery voucher (qdult prison). The characteristics of the participants will be compared with those of the total population of the prison to see whether the final samples differ significantly from the target population.

3.4 Withdrawal and discontinuation

Each participant will be informed that participation is voluntary and that he/she may withdraw from the study at any time. The investigators will not remove any participant from the study. In case of withdrawal during the study, the data already collected will be destroyed.

[1] Deviation from protocol: Recruitment took longer than expected, as it was disrupted by the SARS-CoV-2 pandemic and partial lockdowns.

4 STATISTICS AND METHODOLOGY

4.1. Statistical analysis plan

4.1.1 Sample size calculation

With alpha=.05, power=.80, and an estimated acceptance percentages of 50% for the paper-based material and 70% for the video, we need a sample size of n=190 (n=95 in each group) to detect a statistically significant difference at p=.05 between the two methods in the adult prison (11).
No sample size was computed for the juvenile detention center, as this prison was included for exploratory purposes. In case of lack of power (assessed with sensitivity power calculations), the study can be extended until the required power is obtained.

4.1.2 Analytical strategy

The following analyses will be carried out separately in each prison:
An acceptance rate (with 95% confidence interval) will be calculated (Q1). Acceptance rates will be calculated separately for the groups (Q3).
Bivariate and multivariable analyses will be conducted to test the relationship of the primary outcome with socio-demographic and health variables, in order to identify the profile of those who refuse to give consent (logistic regressions) (Q2).
Finally, responses to questions on consent itself will be compared by acceptance (Q1) and group (Q3). ANOVAs or linear regressions will be used.
Data will be analyzed using Stata or R.

4.2. Handling of missing data

Preliminary analyses to examine the distributions of variables and patterns of missing values will be conducted. To minimise missing values, the questionnaire questions will be asked directly by the interviewers and participants will be asked to answer all questions.
Missing data will not be imputed. If needed, comparisons with the primary outcome (giving or not giving consent) will be made (t-tests or Chi-square tests).

5 REGULATORY ASPECTS AND SAFETY

5.1 Local regulations / Declaration of Helsinki

This research project will be conducted in accordance with the protocol, the Declaration of Helsinki [3], the principles of Good Clinical Practice, the Human Research Act (HRA) and the Human Research Ordinance (HRO) [1] as well as other locally relevant regulations. The Project Leader acknowledges his responsibilities as both the Project Leader and the Sponsor.

5.5 Amendments

Substantial changes to the project set-up, the protocol and relevant project documents will be submitted to the Ethics Committee for approval according to HRO Art. 18 before implementation. Exceptions are measures that have to be taken immediately in order to protect the participants.

5.6 End of project

Upon project termination, the Ethics Committee is notified within 90 days. The electronic anonymized data will be kept for 10 years in the Division of prison health.

6 FURTHER ASPECTS

6.1 Overall ethical considerations

This study will investigate whether general consent for research is feasible in the Division of prison health. General consent for research will encourage research on detained persons by facilitating access to their medical data in order to study and reduce health disparities, for this population with multiple somatic and psychiatric comorbidities.

More specifically, the expected benefits of the project are similar to those expected by the general consent for research at the HUG:
- Enabling a stronger partnership with the patient, especially important for a population with multiple somatic and psychiatric comorbidities;
- Allow access to large samples, thus allowing epidemiological research essential for a better understanding of the characteristics and health needs of detained persons;
- Allow better control and compliance with legal standards regarding the use of data, which is crucial for this vulnerable population.

Moreover, very few - if any - studies document consent in prison, unlike studies in the general population where the characteristics of non-respondents are, if not well known, at least estimable. This project will therefore make it possible to estimate the representativeness of the samples of persons detained in Geneva, by exploring characteristics of persons who do not consent. It will therefore provide an answer to an essential methodological question.
This project will also raise awareness of general consent for research in order to implement it in all Geneva prisons and to obtain the best possible acceptance rate, with the most effective method.

Finally, this project will improve general consent at the HUG, contributing to reducing inequalities in documentation on health status, and ultimately, health inequalities. The video will be made available in all languages at the HUG and will be freely accessible to those who wish to use it.

There are no specific risks for the participants when reading the booklet or watching the video. The questionnaire does not contain any sensitive questions and is quite short. Particular attention will be paid to the voluntary nature of participation in the study, as written informed consent will not be requested at the beginning of the study. Indeed, the signing of a first consent (for the study) could influence the signing of the second one (general consent) and would exclude persons not likely to sign. This could artificially increase the acceptance rate.

6.2 Risk-Benefit Assessment

The risk of adverse consequences for participants is extremely low. No health risks or stress related to the handover are anticipated. There will be no benefit to participants, as the benefits are at the population level (see section 6.1). Given that the risk of adverse consequences is extremely low, we consider the risk-benefit ratio of the study to be appropriate.

6.3 Rationale for the inclusion of vulnerable participants

Detained persons are vulnerable populations. As the project focuses specifically on these populations, it is not possible to obtain data by other means. Participants will be informed that their participation is voluntary, independent of the prison, and that they can withdraw from the study at any time. The study will be conducted in accordance with the ethical principles of prisoner research (12).

7 QUALITY CONTROL AND DATA PROTECTION

7.1 Quality measures

The data will be entered into RedCap using the Internet-connected computer available in the prison medical units, to minimise data entry errors. The interviewers will be trained in data collection and entry. For quality assurance the Ethics Committee may visit the research sites. Direct access to the source data and all project related files and documents must be granted on such occasions.

7.2 Data recording and source data

The questionnaire will be created on RedCap and hosted on the secure server of the HUG. The data will then be exported to Stata for analysis. The information will come from the participants' medical records (for those who consent to the use of their data) and from self-reported questionnaires.

7.3 Confidentiality and coding

Project data will be handled with uttermost discretion and is only accessible to authorized personnel who require the data to fulfil their duties within the scope of the research project. On the CRFs and other project specific documents, participants are only identified by a unique participant number.

7.4 Retention and destruction of study data and biological material

The data will be kept for 10 years before being destroyed.

8 FUNDING / PUBLICATION / DECLARATION OF INTEREST

The project is supported by the University of Geneva (Mimosa funding, 2019).
Results will be published in international peer-reviewed journals and may be presented in international conferences.
Investigators and partners of the project report no conflict of interest.

9 REFERENCES

1. Ordinance on Human Research with the Exception of Clinical trials (HRO).
2. Declaration of Helsinki.
3. Ahalt C, Sudore R, Bolano M, Metzger L, Darby AM, Williams B. "Teach-to-goal" to better assess informed consent comprehension among incarcerated clinical research participants. AMA J Ethics. 2017;19(9):862-72.
4. van Westendorp M. Walk the line [Masterpaper]. Leuven: University of Leuven; 2017.
5. Christopher PP, Garcia-Sampson LG, Stein M, Johnson J, Rich J, Lidz C. Enrolling in clinical research while incarcerated: what influences participants' decisions? Hastings Cent Rep. 2017;47(2):21-9.
6. Ferguson WJ, Cloud D, Spaulding AC, Shelton D, Trestman RL, Altice FL, et al. A call to action: A blueprint for academic health sciences in the era of mass incarceration. J Health Care Poor Underserved. 2016;27(2A):5-17.
7. Baggio S, Fructuoso A, Guimaraes M, Fois E, Golay D, Heller P, et al. Prevalence of attention deficit hyperactivity disorder in detention settings: a systematic review and meta-analysis. Front Psychiatry. 2018;9.
8. Chacowry Pala K, Baggio S, Tran NT, Girardin F, Wolff H, Gétaz L. Blood-borne and sexually transmitted infections: a cross-sectional study in a Swiss prison. BMC Infect Dis. 2018;18(1):539.

9. Gétaz L, Casillas A, Siegrist C-A, Chappuis F, Togni G, Tran N-T, et al. Hepatitis B prevalence, risk factors, infection awareness and disease knowledge among inmates: a cross-sectional study in Switzerland's largest pre-trial prison. J Glob Health.8(2).
10. Chew LD, Griffin JM, Partin MR, Noorbaloochi S, Grill JP, Snyder A, et al. Validation of screening questions for limited health literacy in a large VA outpatient population. Journal of General Internal Medicine. 2008;23(5):561-6.
11. Kelsey JL, Whittemore AS, Evans AS, Thompson WD. Methods in observational epidemiology. Second Edition ed. Oxford, New York: Oxford University Press; 1996 1996/08/08/. 444 p.
12. Pope A, Vanchieri C, Gostin LO. Ethical considerations for research involving prisoners. Washington (DC): National Academies Press (US); 2007 2007.

10 APPENDIX: LANGUAGES SPOKEN IN THE ADULT PRISON

Based on a random selection of 152 medical records made on 22.07.2019, the languages spoken in the adult prison are:

Language	n	%
French	118	77.6
English	12	7.9
Spanish	6	3.9
Italian	5	3.3
Romanian	3	2.0
Albanian	2	1.3
German	2	1.3
Georgian	2	1.3
Russian	1	0.7
Not available	1	0.7

Note: In the case where more than one language is spoken, the most widely spoken has been selected.
We will add Arabic and Portuguese to these languages.
