# Supplementary material for: Comparison of Audiovisual and Paper-Based Materials for 1-Time Informed Consent for Research in Prison: A Randomized Clinical Trial
Source: JAMA Netw Open. 2022 Oct 11;5(10):e2235888. doi: 10.1001/jamanetworkopen.2022.35888 (PMC9554696; doi:10.1001/jamanetworkopen.2022.35888)
Supplement: Supplement 2. — eFigure. Flowchart eAppendix. Information on Informed Consent [file jamanetwopen-e2235888-s002.pdf]

Supplementary Online Content

Baggio S, Gétaz L, Giraudier L, et al. Comparison of audiovisual and paper-based materials for 1-time informed consent for research in prison: a randomized clinical trial. *JAMA Netw Open*. 2022;5(10):e2235888. doi:10.1001/jamanetworkopen.2022.35888

eFigure. Flowchart

eAppendix. Information on Informed Consent

This supplementary material has been provided by the authors to give readers additional information about their work.

© 2022 Baggio S et al. *JAMA Network Open.*

eFigure. Flowchart

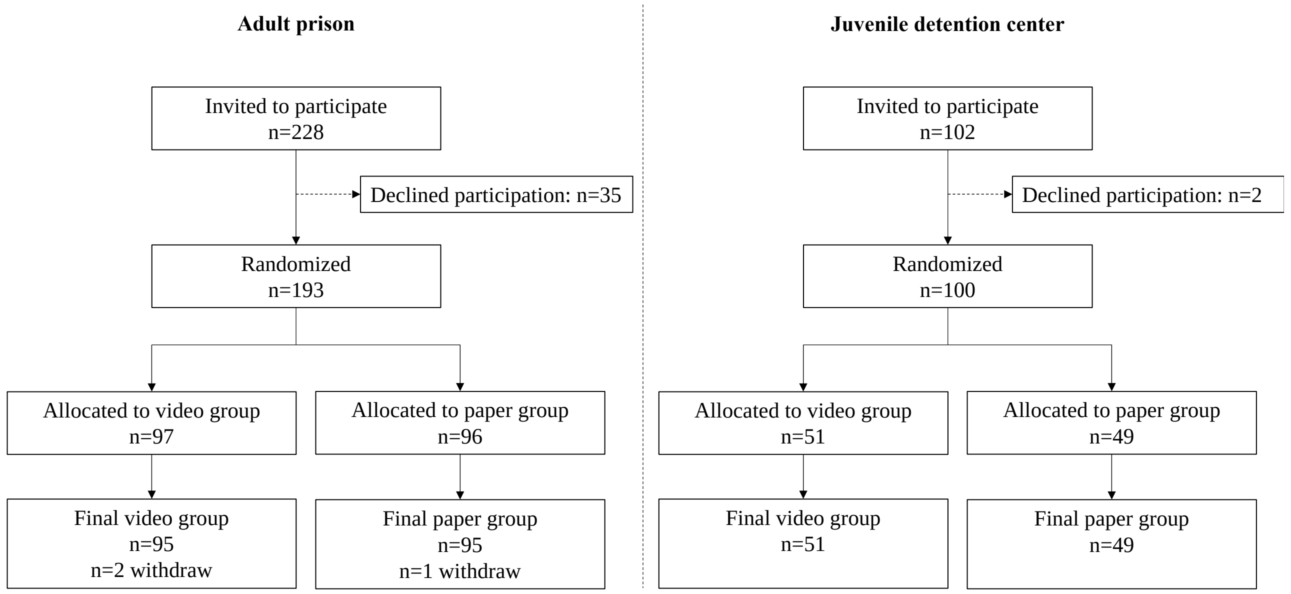

© 2022 Baggio S et al. *JAMA Network Open.*

eAppendix. Information on Informed Consent

Written information for informed consent

Will you be informed about research results?

Research carried out with your samples and data will generally not reveal any individual information for your health. In rare cases, research results might be relevant or significant to your own health and clinical action might be possible. In these cases you might be informed.

Will there be any costs or financial benefit?

There are no additional costs generated. The law excludes commercialization of data and samples. Thus, no financial benefits will be generated for you or the hospital.

\+

INFO

If you have any questions or would like additional information, please contact us at the address below or visit our website at hug-ge.ch/aider-recherche

Aider la recherche • Photo: © Julien Gregorio • Septembre 2019

Anglais

ASSISTING
RESEARCH

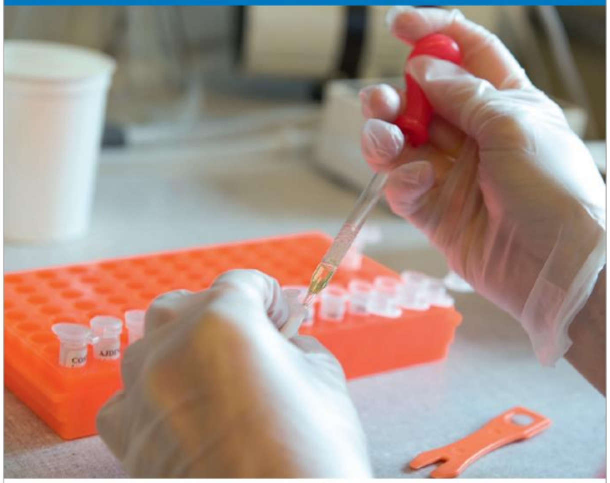

HUG Hôpitaux Universitaires Genève

Information about the use of health-related data and samples for research purposes

Dear Patient

Our ability to diagnose and treat diseases has progressed significantly in recent decades. These progresses are the result of long-standing medical research in which doctors, scientists and patients of several generations have actively participated. An important part of this research relies on patients' health-related data from medical history, such as results of laboratory analyses, therapy information or genetic predispositions. Any biological material collected during the hospital stay and which is no longer needed for the treatment, is also extremely valuable for research. These leftover samples can be for example blood, urine or tissue samples.

This leaflet explains how patients can contribute to medical progress and provides information in terms of data protection and associated rights. Thank you for your interest and attention.

How can you contribute to research?

By signing the declaration of consent with «Yes», you are making your clinical data and leftover samples available for research purposes. Data and samples include those, which have been collected and will be collected during your hospital stay. Your consent is voluntary. It remains valid indefinitely or until withdrawn. You are entitled to withdraw your consent at any time without having to justify your decision. After withdrawal your data and samples will not be available for new projects. Your decision has no effect on your medical treatment.

How are your health-related data and samples protected?

Data are stored within the hospital and protected in accordance with the applicable legal requirements. Only authorised employees from the hospital, e.g. physicians, have access to your uncoded data and samples. Your samples are stored in biobanks which contain structured collections of samples under safety regulations (biobank regulations).

If your data and samples are used for a research project, they will be coded or anonymized. Coded means that all personal information such as your name or birth date is replaced by a code. The key showing which code belongs to which person is kept safe by a professional who is not involved in the research project. Persons who do not have the code are not able to identify you. In case of anonymization, the link between the biological material and/or associated data and the participant is definitely removed, so that no specific participant can be reidentified.

Who may use your health-related data and samples?

Data and samples may be used by authorised researchers for research projects within the hospital or in collaboration with public institutions (such as other hospitals) and private entities (such as pharmaceutical companies), in Switzerland and abroad. For research abroad, it must be ensured that at least the same data protection conditions are followed as in Switzerland. The projects may include genetic analyses for research purposes. Research projects relying on your data and samples have to be authorized by the relevant ethics committee.

Video information for informed consent

Video link:
https://vimeo.com/654887207/fc26d1e957

© 2022 Baggio S et al. *JAMA Network Open.*

Informed consent

Anglais

Declaration of consent for the use of health-related data and samples for research purposes

....

Patient's surname and name | Date of birth

I herewith agree
that my health-related data and samples collected during health care (ambulant or as an inpatient) will be available for research purposes

☐ Yes ☐ No

I understand

- the explanations about further use of my health-related data and samples for research purposes that are detailed in the information sheet.
- that my personal data are protected.
- that my data and samples may be used in national and international projects, within the public and private sectors.
- that projects may include genetic analyses of my samples for research purposes.
- that I may be recontacted in case of individually significant findings, if any.
- that my decision is voluntary and has no effect on my treatment.
- that my decision is not limited in time.
- that I may withdraw my consent at any time without having to justify my decision.

....

Place, date | Patient's signature, if judicious

....

Place, date | Signature of legal representative, if required (Name and relationship to patient)

Please consider the following contact or your physician if you have further questions or if you wish to receive a copy of this form with signature.

Hôpitaux Universitaires Genève

Patient label

Questionnaire

© 2022 Baggio S et al. *JAMA Network Open.*

Understanding consent

	True	False	Do not known
1. My name is kept with my data.			
2. My decision is valid indefinitely.			
3. I must justify my decision if I refuse to participate.			
4. My data can be sold.			
5. My data may be used elsewhere than in Geneva.			
6. I can withdraw my consent at any time.			
7. If I accept, the doctors will do unnecessary additional tests for my medical follow-up.			
8. Only authorized scientists will have access to my data.			

Consent assessment

	1	2	3	4	5	6	
1. The consent is **difficult** to understand.							The consent is **easy** to understand.
2. The information provided is **not sufficient**.							The information provided is **sufficient**.
3. You did **not feel free** to sign the consent.							You **felt completely free** to sign the consent.
4. **You feared** that your decision would affect your **access to care**.							You **did not fear at all** that your decision would affect your **access to care**.
5. **You feared** that your decision would influence your **legal proceedings**.							You **did not fear at all** that your decision would influence your **legal proceedings**.

© 2022 Baggio S et al. *JAMA Network Open.*

Clinical questions

1. Have you ever consulted a psychologist or a psychiatrist in prison?

- yes
- no

2. Have you ever consulted a psychologist or a psychiatrist before your incarceration?

- yes
- no

3. Do you have any somatic disease?

- yes
- no

4. If yes, which ones?
_ _
_ _

5. Do you have any psychiatric disease?

- yes
- no

6. If yes, which ones?
_ _
_ _

7. Do you have ongoing medical treatments?

- yes
- no

8. If yes, which ones?
_ _
_ _

Health literacy

1: never 2: seldom 3: sometimes 4: often 5: very often 6: always

	**1**	**2**	**3**	**4**	**5**	**6**
1. How often do you have problems understanding about your medical condition because of difficulty reading hospital materials?						
2. How often do you have someone help you read hospital materials?						
3. How confident are you filling out forms by yourself?						

© 2022 Baggio S et al. *JAMA Network Open.*

Sociodemographic questions

1. Age: _ _ years old

2. Nationality: _ _ _ _ _ _ _ _ _ _ _ _ _ _ _ _

3. Country of origin: _ _ _ _ _ _ _ _ _ _ _ _ _ _ _ _

4. Year arrived in Switzerland: _ _ _ _

5. Residence permit in Switzerland:
   - Swiss passport
   - Permit B, C or G
   - Permit F or N
   - No permit
   - Passport from a country in Western Europa

6. Age when stopped school: _ _ years old

7. Level of education:
   - never attended school
   - incomplete primary school
   - finished primary school
   - incomplete high school
   - finished high school
   - incomplete college / baccalaureate
   - finished college / baccalaureate
   - technical training after high school
   - university

8. Health insurance coverage before detention:
   - yes
   - no
   - do not know

9. Kind of health insurance coverage:
   - LaMal (Switzerland)
   - health insurance of another country, which: _ _ _ _ _
   - other: _ _ _ _ _ _ _ _ _ _ _ _ _ _ _ _ _ _ _

10. Date prison entry: _ _ / _ _ _ _

© 2022 Baggio S et al. *JAMA Network Open.*
