# Supplementary material for: Comparison of Audiovisual and Paper-Based Materials for 1-Time Informed Consent for Research in Prison: A Randomized Clinical Trial
Source: JAMA Netw Open. 2022 Oct 11;5(10):e2235888. doi: 10.1001/jamanetworkopen.2022.35888 (PMC9554696; doi:10.1001/jamanetworkopen.2022.35888)
Supplement: Supplement 3. — Data Sharing Statement [file jamanetwopen-e2235888-s003.pdf]

Data Sharing Statement

Baggio. Comparison of Audiovisual and Paper-Based Materials for 1-Time Informed Consent for Research in Prison. *JAMA Netw Open*. Published October 11, 2022. doi:10.1001/jamanetworkopen.2022.35888

Data
Data available: No

Additional Information
Explanation for why data not available: The datasets generated and/or analysed during the current study are not publicly available due their sensitive nature (detained persons) but are available from the corresponding author on reasonable request.
